# Clitoridectomy

**Published:** 1895-08

**Authors:** 


### CLITORIDECTOMY.

Dr. Clara Burrus, of the Ogdenburg Insane Hospital, remarks (*Amer. Jour. of Ins.*) that hardly any condition among the insane is so deplorable in its manifestations as masturbation. It occurs in women, by nature and training refined and cultured, and is often accompanied by incredible obscenity of thought, speech and manner. Hypertrophy of the nymphæ or of the clitoris are said to be signs of masturbation, yet these anomalies are often found in non-masturbatory cases. In many cases the perversion is found in patients with normal vulvæ, where the nymphæ and clitoris are very small or where they are practically absent. Repeated excitation of the genitalia by masturbation probably results in hyperæmia of the external genitalia and the uterus and its adnexa. Hyperæmia frequently induced leads to congestion and congestion to hypertrophy as a result of the exaggerated nutrition of these organs. But just why this hypertrophy should not result in all masturbatory cases it is difficult to determine. Sometimes hypertrophy of one nymphæ only is found. The secretion of the praeputium clitoridis is often a hardened semi-organized mass, so hard sometimes as to merit the term concretion; the mass being held there by a sort of superficial prepuce adhesion, in many cases removed with difficulty. That this should give rise to nervous irritation which would in many cases lead to pruritus and subsequently to masturbation is not surprising. "Orificial" enthusiasts would probably assert that masturbation when present is the direct result of this condition alone. From the anatomical structure of this organ and its nerve supply it is obvious that secretion retained as above described may exert a pernicious influence on the female economy. The removal of such secretion and the destruction of the adhesions naturally lead to hope of ameliorating the mental condition. While this condition is often associated with masturbation, it also occurs in cases where the habit does not exist. The condition may, however, have given rise to other nervous disturbances quite as pernicious, if not to masturbation itself. In dementia amelioration of the mental disorder cannot be expected even though the condition be removed. At the same time it would be an unjustifiable neglect to fail to remove when possible a condition which may exert a pernicious influence on the nervous system. Not so the condition of clitoris elongation, the operation for the removal of which is so much in vogue. It seems a very reprehensible practice. The worst case of masturbation Dr. Burrus has seen is that of a young woman who has been clitoridectomized. This patient masturbated more or less all her life. After several attacks of nymphomania she decided to have the clitoris amputated. The operation increased the severity of the nymphomania, causing shameless—almost literally continuous—self-abuse. Some cases will persist in the habit in spite of everything that can be done—padded mitts, restraining of hands and feet, the protection sheet, moral suasion. All means are tried to protect the patient from herself with the result of seeing her when thwarted exhibit manifestations which show that she is indulging in a sort of vicarious mental masturbation, the center of excitement apparently being psychical.—*Medical Standard.*

